# Visible-Light-Active Black TiO_2_ Nanoparticles with Efficient Photocatalytic Performance for Degradation of Pharmaceuticals

**DOI:** 10.3390/nano12152563

**Published:** 2022-07-26

**Authors:** Luminita Andronic, Daniela Ghica, Mariana Stefan, Catalina Gabriela Mihalcea, Aurel-Mihai Vlaicu, Smagul Karazhanov

**Affiliations:** 1 Product Design, Mechatronics and Environment Department, Transilvania University of Brasov, Eroilor 29, 500036 Brasov, Romania; 2 National Institute of Materials Physics, Atomistilor 405A, 077125 Magurele, Romania; ghica@infim.ro (D.G.); catalina.mihalcea@infim.ro (C.G.M.); amvlaicu@infim.ro (A.-M.V.); 3 Faculty of Physics, University of Bucharest, Atomistilor 405, 077125 Magurele, Romania; 4 Solar Energy Department, Institute for Energy Technology, P.O. Box 40, NO-2027 Kjeller, Norway; smagul.karazhanov@ife.no

**Keywords:** black TiO_2_, defective TiO_2_, Ti^3+^ states, chemical reduction, photocatalysis, amoxicillin, wastewater treatment

## Abstract

Special attention has recently been paid to surface-defective titanium dioxide and black TiO_2_ with advanced optical, electrical, and photocatalytic properties. Synthesis of these materials for photodegradation and mineralization of persistent organic pollutants in water, especially under visible radiation, presents interest from scientific and application points of view. Chemical reduction by heating a TiO_2_ and NaBH_4_ mixture at 350 °C successfully introduced Ti^3+^ defects and oxygen vacancies at the surface of TiO_2_, with an increase in the photocatalytic degradation of amoxicillin—an antibiotic that is present in wastewater due to its intense use in human and animal medicine. Three TiO_2_ samples were prepared at different annealing temperatures to control the ratio between anatase and rutile and were subjected to chemical reduction. Electron paramagnetic resonance investigations showed that the formation of surface Ti^3+^ defects in a high concentration occurred mainly in the anatase sample annealed at 400 °C, contributing to the bandgap reduction from 3.32 eV to 2.92 eV. The reduced band gap enhances visible light absorption and the efficiency of photocatalysis. The nanoparticles of ~90 m^2^/g specific surface area and 12 nm average size exhibit ~100% efficiency in the degradation of amoxicillin under simulated solar irradiation compared with pristine TiO_2_. Mineralization of amoxicillin and by-products was over 75% after 48 h irradiation for the anatase sample, where the Ti^3+^ defects were present in a higher concentration at the catalyst’s surface.

## 1. Introduction

Photocatalysis is becoming more and more attractive in industrial water decontamination [1,2]. The challenges are primarily related to the low efficiency of photocatalysts under the visible solar spectrum, the short lifetime of electrons and holes due to their recombination, and the short lifespan of the photocatalyst [3]. The applications of TiO_2_ in solar energy conversion and environmental technologies are especially hindered by the inefficient use of solar energy, due to its wide bandgap of ~3.2 eV for anatase and ~3.0 eV for rutile in its crystallinity. The first generation of photocatalysts was overused in heterogeneous photocatalysis between 1980 and 2000 [4,5].

Researchers have focused on expanding the response of TiO_2_ to the visible light region. They have conducted intensive studies on materials obtained by doping TiO_2_ with metals and non-metals, representing the beginning of the second generation of photocatalysts [6,7] and extending the adsorption edge of the TiO_2_ in the visible region [8]. It was found that doping either shrinks the bandgap energy (E_g_) or creates mid-gap states in metal-doped TiO_2_, enhancing the electron–hole separation rate [9]. For example, the bandgap energy of Sn^4+^-doped TiO_2_ decreased from 3.10 to 3.00 eV [10]. Cobalt-doped TiO_2_ with 2% cobalt had an indirect bandgap reduced at 1.93 eV [11]. Cu doping enhanced the photocatalytic antimicrobial activity of TiO_2_ under visible light irradiation by creating oxygen vacancies by substituting Ti^4+^ ions with Cu^2+^ ions in the TiO_2_ lattice and decreasing the bandgap at 2.8 eV [11]. Fe-doped TiO_2_ showed a red shift and a bandgap of 2.6 eV when the impurity concentration was 4 mol %, improving the prepared materials’ optical properties [11].

If metal or non-metal dopants shift the valence band or conduction band edge, the Ti^3+^ defects and oxygen vacancies narrow the bandgap and modify the crystal color. As a result, white titanium oxide transforms into blue-, grey-, and black-colored TiO_2_ [12].

Because of its tunable bandgap and surface disorder [13], the presence of point defects such as Ti interstitial, Ti^3+^, surface oxygen vacancies, and the fast electron–hole separation [14], the black TiO_2_ is a more attractive visible light photocatalyst than white TiO_2_. Over the past ten years, it has become attractive for anode applications in electrochemical pollutant degradation from wastewater under sunlight conditions [15] for solar energy conversion [16], for cathode applications for lithium–sulfur batteries as the next generation of electrochemical energy storage systems [17,18], and for electron transporter layers in planar perovskite solar cells [19] and supercapacitors [20].

Ansari et al. [8] obtained defective titanium oxide using a commercial TiO_2_ (size < 25 nm) powder. The surface defects created under ambient conditions narrowed the bandgap from 3.15 eV to 2.85 eV. In addition, the defective TiO_2_ provided three-fold increased photodegradation efficiency of methylene blue compared with pristine white TiO_2_.

The black TiO_2_ also has numerous applications in the gas phase: photo-assisted catalytic degradation of air pollutants [21], photocatalytic CO_2_ reduction [22], hydrogen production [23], solar fuel generation [24], oxygen sensor-based on colorimetric effect between white and black TiO_2_ [25], and propane dehydrogenation for propylene production [26].

Numerous synthesis methods have been developed in the past for black TiO_2_. The majority of them are based on high-temperature processing in various reducing atmospheres such as nitrogen, vacuum, argon, or a combination of them [27]; in pulsed laser ablation conditions [28]; and plasma processing under ambient conditions [29]. However, the synthesis methods focus on chemical reduction methods, which are scalable. The chemical reduction of pristine TiO_2_ with NaBH_4_ has some advantages over other methods, as it can be done at a relatively low temperature of ~350 °C under atmospheric conditions or in an oxygen-poor atmosphere. The generation of Ti^3+^ defects and oxygen vacancies is responsible for the enhanced photoactivity and can be controlled by the NaBH_4_:TiO_2_ ratio [30]. NaBH_4_ can reduce Ti^4+^ to Ti^3+^ according to Equations (1) and (2), H_2_ is formed in situ at room temperature (Equation (3)), and white TiO_2_ turns black [31]. On the other hand, the chemical reduction could introduce sodium and boron as trace impurities provided by the reductant agent [32].

(1) $${\mathrm{NaBH}}_{4}+8{\mathrm{OH}}^{-}\to {\mathrm{NaBO}}_{2}+8e^{-}+6H_{2}O$$

(2) $${\mathrm{Ti}}^{4+}+e^{-}\to {\mathrm{Ti}}^{3+}$$

(3) $${\mathrm{BH}}_{4}^{-}+2H_{2}O\to {\mathrm{BO}}_{2}^{-}+4H_{2}$$

In this work, we have investigated the importance of the TiO_2_ structure in the distribution of the Ti^3+^ defects created by using sodium borohydride as a reductant in an oxygen-poor atmosphere at lower temperatures in nanocrystalline TiO_2_ powders with different structures (anatase, rutile, or a mixture of the two phases). Furthermore, the photocatalytic activity evaluation proved that the surface defect concentration plays a key role in heterogeneous photocatalysis.

## 2. Materials and Methods

### 2.1. Synthesis of Black TiO_2_ Nanoparticles

The synthesis of black TiO_2_ nanoparticles involves a two-step process. In the first step, TiO_2_ sol-gel was obtained from titanium isopropoxide (TTIP) (≥97.0%, Sigma-Aldrich) as a Ti precursor in nitric acid medium and annealed at different temperatures—at 400 °C (sample T400W), 550 °C (sample T550W), and 800 °C (sample T800W)—for three hours, to ensure a proper anatase:rutile ratio. The synthesis method was reported in our previous work [33,34]. In the second step, the white TiO_2_ powder was turned into black powder using NaBH_4_ (sodium borohydride, 99%, VenPure) as a chemical reductant; the mass ratio TiO_2_:NaBH_4_ was kept at 2:1 for all samples, having already been optimized in our previous experiments [35]. The powders were homogenized by mixing for 10 min in a mortar, then wrapped in aluminum foil with a minimum of air inside and placed in a calcination oven at 350 °C for 1 h (increasing the temperature at 5 °C/min). The reaction mixture was cooled in the oven to room temperature, washed with HCl 1M solution, filtered several times until the pH reached around 6–7, and dried in an oven for 12 h at 105 °C. As a result, three black powders were obtained T400B, T550B, and T800B.

### 2.2. Photocatalysts Characterization

X-ray diffraction (XRD) measurements were performed using a Rigaku SmartLab XE diffractometer in the Bragg–Brentano configuration with a copper anticathode X-ray generator. Rietveld structural analysis was performed with the Fundamental Parameter Approach using TOPAS 2.4 [36]. 

The samples’ morphology and structure were analyzed using a JEOL JEM-2100 transmission electron microscope, equipped with an X-ray Energy Dispersive Spectroscopy (EDS) detector used for elemental analysis. The samples were prepared by dispersing the powder in ethanol and drop-casting on the TEM grids.

The particle size distribution, ranging from 0.6 nm to 10 µm, was analyzed using the dynamic light scattering (DLS) method investigated by Malvern Zetasizer Nano ZS (HeNe laser, 633 nm; angle, 173°; polystyrene cuvette, 2.5 mL). First, a suspension of 10 mg nanoparticles into 10 mL ultrapure water was sonicated for 5 min prior to measuring the size of the particles. Then, three replicate samples per time point were performed; the data are shown as mean values. The resulting intensity of the scattered light is distinct from the particle size that the laser beam encountered and is detected by signal readers. Therefore, the data for DLS is represented as intensity frequency (%).

Nitrogen adsorption–desorption isotherms were acquired at liquid N_2_ temperature (−196 °C) on a Micromeritics Tristar II Plus instrument. Before analysis, the powders were outgassed overnight at 110 °C under a vacuum (10^−3^ Pa). BET-surface areas were calculated using the BET equation, whereas mean pore size, pore size distribution and pore volume were estimated using the Barrette–Joyner–Halenda (BJH) method. In addition, the mean nanoparticle size was determined.

Electron paramagnetic resonance (EPR) investigations were performed in the 90 K–295 K temperature range in the Q (34 GHz) microwave frequency band. The T550B and T800B samples were inserted in calibrated pure fused-silica tubes with 1 mm i.d., while the other samples were inserted into tubes with 2 mm i.d. The EPR measurements were carried out with a Bruker E500Q spectrometer equipped with an ER5106QT/W resonator and a CF935 continuous flow cryostat from Oxford Instruments. The derivative dP/dB of the microwave power P absorbed by the sample was recorded as a function of the static magnetic field B. In order to improve the signal-to-noise ratio and evidence of low-intensity EPR signals, some of the spectra were recorded with multiple scans (up to 20 scans), which led to the enhancement of the background resonator signals as well, such as a Mn^2+^ spectrum or a broad line at ~1185 mT. The EPR spectra analysis and simulation and the determination of the EPR parameters were performed with the EasySpin v. 5.2.33 program [37].

The optical properties and the bandgap change were investigated by diffuse reflectance measurements using a diffuse reflectance–UV/visible spectrophotometer (DRS) (Ocean Optics QE65000) in the wavelength range of 200–1000 nm.

### 2.3. Photocatalytic Experiments

Amoxicillin (AMX) was used as a pollutant model to investigate the photocatalytic activity of black TiO_2_. A higher concentration than that usually found in wastewater was used to investigate the mineralization and photocatalysis mechanism.

The photocatalytic activity of the synthesized catalysts was investigated in a batch reactor under simulated visible light. The irradiation sources consisted of two fluorescent tubes (F18W/T8, UVA, 340–400 nm, λ_max_ = 365 nm, flux intensity 3Lx) and six white visible tubes (TL-D Super 80 18 W/865, VIS, 400–700 nm, with λ_max_ = 565 nm, flux intensity 28Lx). The aluminum box ensures uniform light reflection, and four tubes are located above, with two on the left and two on the right. A fan ensured a constant temperature of 26 °C throughout the experiments. In addition, the quartz containers were closed to prevent evaporation of the solutions.

In each experiment, a volume of 50 mL of amoxicillin (20 ppm concentration) was mixed with the synthesized catalyst (0.6 g/L) and stirred (200 rpm) in the dark for 30 min to establish adsorption–desorption equilibrium. After 30 min, the light was turned on, and 500 μL aliquots were taken at pre-set time intervals and filtered (Millex PES 0.45 μm filter) to remove the photocatalyst.

The residual AMX concentration was analyzed using High-Performance Liquid Chromatography with UV detection (HPLC-UV) at the maximum absorption wavelength of 254 nm and a low-pressure gradient. The separation was achieved using a Nucleosil 100-5 C18 column (Macherey-Nagel, 5 µm particle size, length 2 50 mm, i.d. 4.6 mm) at 40 °C. The mobile phase consisted of 85% KH_2_PO_4_ adjusted at pH = 5 with a KOH 45% *w/w* solution and 15% methanol. The injection volume was 20 µL with a 1.5 mL/min flow rate. The retention time was 3.06 min.

Amoxicillin mineralization was evaluated by a total organic carbon (TOC) method using a TOC analyzer (Shimadzu TOC-VCSN). Before analysis, the samples were centrifugated at 4000 rpm and filtered by Millex PES 0.20 um filters.

## 3. Results and Discussion

### 3.1. Structural and Morphological Characterization

#### 3.1.1. X-ray Diffraction

**Figure 1:**
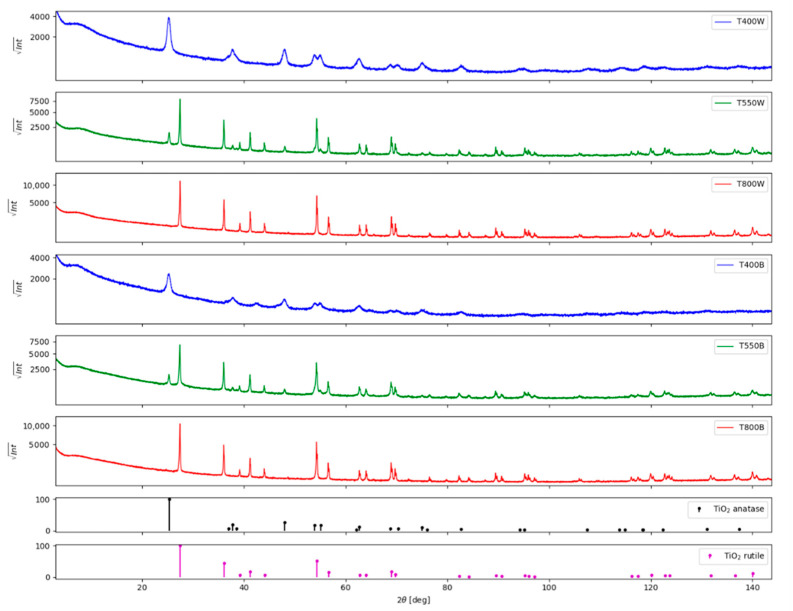
XRD patterns of white and black titanium oxide samples.

The TiO_2_ white samples prepared by sol-gel show a gradual phase transformation with increasing annealing temperature from anatase (*I*4_1_/amd space group) at 400 °C to rutile at 800 °C (I4_2_/mnm space group) through a mixture of the two phases at 550 °C, with rutile being the main component (84% weight). As the annealing temperature increases, the volume averaged crystallite size increases from 15 nm for anatase at 400 °C to 45 nm at 550 °C and for rutile from 127 nm at 550 °C to 130 nm at 800 °C. This can be observed as the diffraction peaks become sharper with increasing annealing temperature (Figure 1). Furthermore, besides anatase and rutile, one can observe quasi-amorphous phases in the diffraction peaks in the 2θ range 8–12°. An unidentified residual phase was also observed for the black TiO_2_ sample annealed at 400 °C, with 2θ peaks at 43° and 65°. Another peculiarity is the slight change in the tetragonality ratio (a/c) for anatase: the unit cell is slightly expanded along the (a) axis for T400W compared to T550W and slightly compressed along with the (c) axis for T400W compared to T550W (see actual values in in Table S1, Supplementary Materials). The results are consistent with those reported by Tian and Grecu [13,38]. However, a definite explanation for this phenomenon has yet to be determined [13].

After chemical reduction, the black samples show modifications of the crystalline parameters. The volume averaged crystallite size decreases from 15 nm to 12 nm for anatase at 400 °C, from 45 nm to 39 nm for the anatase phase of T550B, from 127 nm to 75 nm for the rutile phase of T550B, whereas it remains unchanged for rutile in T800B at 130 nm. The anatase tetragonality ratio (a/c) is further affected by a slight expansion along with the (c) axis for T400B compared to T550B.

#### 3.1.2. Transmission Electron Microscopy

**Figure 2:**
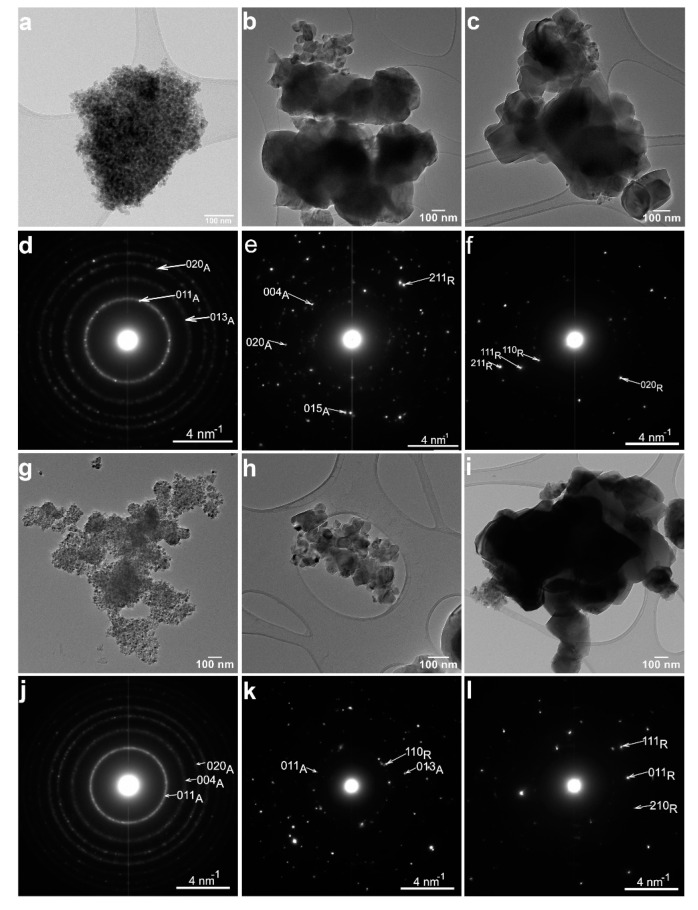
Low-magnification TEM images and the corresponding electron diffraction patterns for the white samples: (**a**,**d**) T400W, (**b**,**e**) T550W, (**c**,**f**) T800W; for the black samples: (**g**,**j**) T400B, (**h**,**k**) T550B, (**i**,**l**) T800B. The letter “A” from the indexed diffraction patterns stands for “anatase”, while the letter “R” stands for “rutile”.

The structural properties of the six samples were further confirmed by TEM (Figure 2). The sample morphology is similar, and the nanoparticle size is comparable for each pair of white-black samples. However, the size of the nanoparticles significantly increases when increasing the annealing temperature from 400 °C to 800 °C for both white (Figure 2a–c) and black samples (Figure 2g–i). According to the electron diffraction patterns from Figure 2d,j, the structure of both T400W and T400B samples is anatase. The electron diffraction patterns of the T550W (Figure 2e) and T550B (Figure 2k) samples reveal anatase and rutile crystallographic phases. The T800W (Figure 2f) and T800B (Figure 2l) samples have a rutile structure.

In the case of the T550W sample, the low-magnification TEM image in Figure 2b shows two distinct nanoparticle populations, with mean sizes of about 50 nm and over 100 nm, respectively. The electron diffraction patterns acquired separately from areas corresponding to the two differently sized nanoparticles showed that the structure of the larger nanoparticles is rutile, while that of the smaller nanoparticles is anatase (see Figure S1 Supplementary Materials).

**Figure 3:**
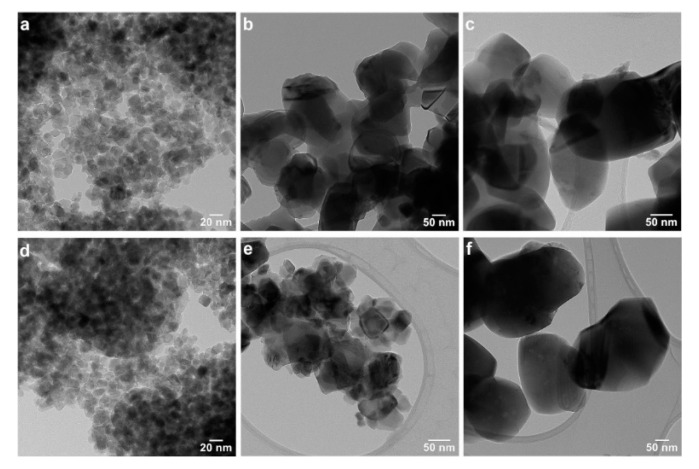
TEM images at higher magnifications, revealing the samples’ morphology: (**a**) T400W, (**b**) T550W, (**c**) T800W, (**d**) T400B, (**e**) T550B, and (**f**) T800B.

**Figure 4:**
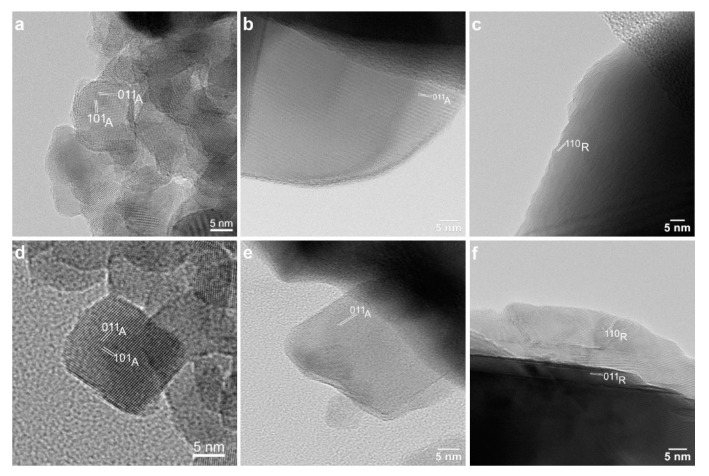
HRTEM images of the (**a**) T400W, (**b**) T550W, (**c**) T800W, (**d**) T400B, (**e**) T550B, and (**f**) T800B samples. Interplanar distances are marked for the anatase (**a**,**b**,**d**,**e**) and rutile (**c**,**f**) phases.

The nanoparticle morphology was further investigated by TEM at higher magnifications (Figure 3) and in the high-resolution mode (HRTEM) (Figure 4). The morphology of all the samples is rather isotropic, with a tendency of faceting. In the T400W and T400B samples, the observed nanoparticle sizes range from 8 to 26 nm in the first and 7 to 20 nm in the second sample (Figure 3a,d). With the annealing temperature increasing from 400 °C to 550 °C, the nanoparticles’ dimension visibly increases, up to 130 nm in T500W and 70 nm in T500B, without a notable morphology change. The interplanar distances measured in the HRTEM images of the T550W (Figure 4b) and T550B (Figure 4e) samples confirm the anatase structure of the smaller nanoparticles. In the case of the T800W and T800B samples, the nanoparticles are very large, ranging from 170 to 260 nm.

The EDS investigation of the elemental composition of the six samples revealed the absence of intrinsic impurities in the quantification limit (Figure S2, Supplementary Materials).

#### 3.1.3. Particle Size Distribution 

The particle size distribution is shown in Figure S3 (Supplementary Materials). For particles of similar size, the polydispersive index (PDI) must be less than 0.3–0.4; higher values indicate the presence of particles of different sizes [39]. The suspension consists of TiO_2_ nanoparticles with an average diameter of 1.8 µm, 1 µm, and 380 nm for the T400W, T550W, and T800W samples, respectively. This might be due to the hydrophilic character of TiO_2_ powders, leading to the adherence of water molecules to the surface of TiO_2_ and the resulting agglomeration of nanoparticles. The agglomeration is higher for the T400W powder due to the larger BET surface, favoring the adsorption of pollutant molecules and, consequently, a higher photocatalytic efficiency. For the white TiO_2_ powders, the PDI indexes are 0.3–0.48, 0.58–0.68, and 0.28–0.36 for the T400W, T550W, and T800W samples, respectively.

The DLS measurements of the black T400B, T550B, and T800B samples resulted in lower mean diameters of 330 nm, 360 nm, and 410 nm, respectively, than for the white TiO_2_ samples. The PDI index is less than 0.4 for all black TiO_2_ samples, suggesting the presence of almost uniform particles. In addition, the DLS measurements revealed a larger particle size for all samples compared to the TEM results due to the agglomeration.

#### 3.1.4. Brunauer–Emmett–Teller (BET) Investigations 

The BET surface area, pore volume, and pore diameter of the synthesized samples were obtained from N_2_ adsorption–desorption measurements and are summarized in Table 1. The BET surface area and pore volume decreased from 89.08 m^2^/g to 2.41 m^2^/g and from 0.235268 to 0.001183 cm^3^/g, respectively, when increasing the calcination temperature from 400 to 800 °C. This can be explained by the loss of organic residues from the samples, which negatively influences the photocatalytic activity. On the other hand, the reduction of the surface area induced by the annealing at higher temperatures can also be due to the increase in particle size and the agglomeration of the TiO_2_ powders. Indeed, the pore size distribution of the raw TiO_2_ produced by the sol-gel method and of the calcinated samples at different temperatures was calculated using the BJH method (Table 1). The materials presented a mesopore width distribution between 13.15 and 2.15 nm. Furthermore, in the white TiO_2_ powders, the pore size increases from 7.49 to 13.15 nm when the calcination temperature increases from 400 to 550 °C, and decreases from 13.15 to 2.15 nm when the temperature increases to 800 °C. These results could be due to particle aggregation, sintering to form pores of smaller sizes.

In the black-colored powders, the reduction process with NaBH_4_ did not change the specific BET surface area of the powder. Furthermore, the particle size is only 66–67 nm for the T400W and T400B powders, increasing from 845 nm for sample T550B to 1552 nm for sample T800B. The small size particles present in the T400W and T400B powders are responsible for the high photocatalytic efficiency in the photodegradation process of amoxicillin.

The N_2_ adsorption–desorption isotherms displayed in Figure S4 (Supplementary Materials) show the characteristic features corresponding to a type IV isotherm according to IUPAC classification [40], specific for adsorption on the mesoporous substrate with pores between 2 and 15 nm. The T400W and T400B isotherms show an H3 hysteresis loop. For the T550W, T550B, T800W, and T800B samples, type H1 narrow hysteresis loops—with the adsorption curves being almost coincident with the desorption curves and increasing rapidly at p/p_0_ = 0.9—were observed.

### 3.2. Spectroscopic Characterization

#### 3.2.1. Optical Properties

Reflectance spectra of the T400W, T400B, T550W, T550B, T800W, and T800B samples were measured by optical instruments. First, diffuse reflectance spectra (DRS) were recorded in the 200–1000 nm range, and then the direct bandgap E_g_ was determined by representing the Kubelka–Munk function versus energy (measured in eV) (Figure S5, Supplementary Materials). A net decrease in E_g_, induced by the chemical reduction, was observed: from 3.32 eV (sample T400W) to 2.92 eV (sample T400B) and from 3.08 eV (sample T550W) to 2.96 eV (sample T550B). On the other hand, sample T800B showed no change in E_g_ compared to T800W.

#### 3.2.2. Electron Paramagnetic Resonance

**Figure 5:**
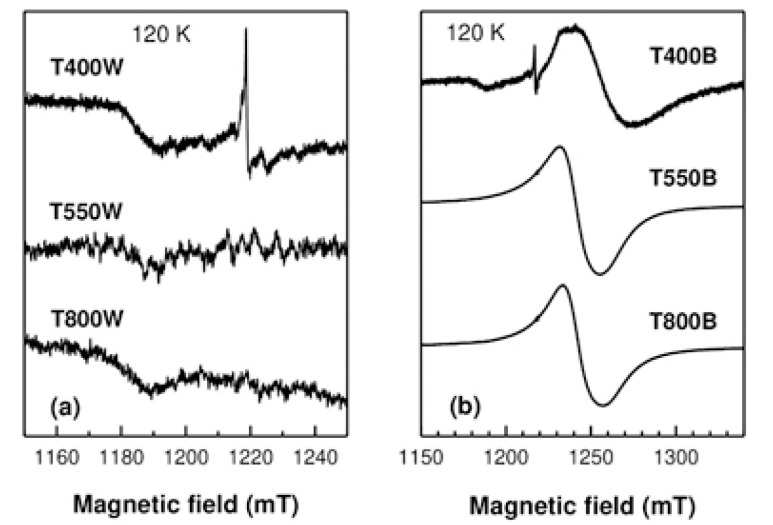
Q-band EPR spectra of the white (**a**) and black (**b**) samples at 120 K. The broad line at ~1185 mT in (**a**) is a background resonator signal.

EPR spectra consisting of very weak lines in the g ~ 2 region (around 1220 mT) were observed at room temperature only for the T400W and T400B samples. Below 200 K, a broad signal appeared in the spectra of all black samples, growing in intensity as the measuring temperature was further decreased. No EPR signal was detected for the T550W and T800W samples at any temperature. Figure 5 displays the EPR spectra recorded for all white (Figure 5a) and black (Figure 5b) samples at 120 K. The broad intense line observed in the black samples is, as further shown, in the spectral region of the Ti^3+^ centers, and it is a net effect of the chemical reduction.

**Figure 6:**
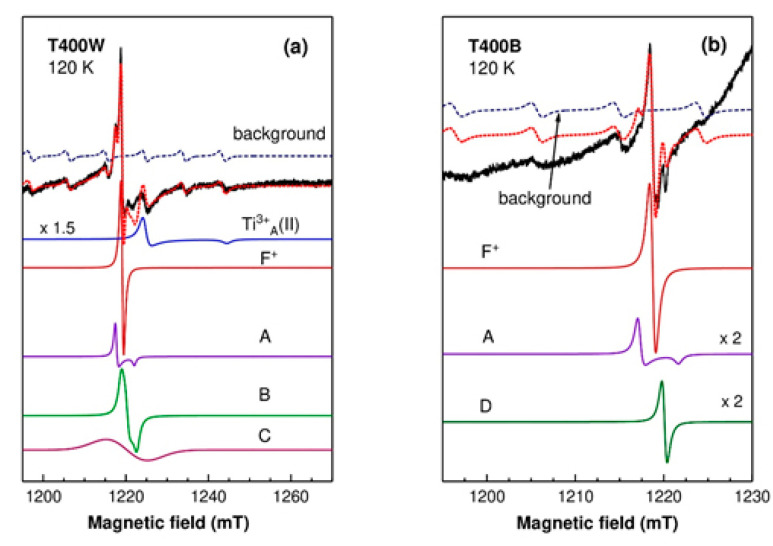
Experimental (solid black line) spectra of the T400W (**a**) and T400B (**b**) samples at 120 K. The simulated spectra (red dotted line) are the sums of the calculated spectra of the various paramagnetic centers represented below. The background resonator Mn^2+^ spectrum (navy blue dashed line) was included for accuracy. The amplitudes of the Ti^3+^_A_(II) (**a**) and A, D (**b**) spectra were multiplied with the factors from the figure for better observation.

Figure 6 displays the g ~ 2 region of the T400W and T400B EPR spectra recorded at the same temperature of 120 K. The T400W spectrum (Figure 6a) is dominated by an isotropic line at g = 2.0028 with a peak-to-peak linewidth of 0.7 mT, attributed to the F^+^ centers, consisting of a singly charged oxygen vacancy [38,41]. Several other lower-intensity lines observed in the T400W spectrum were assigned to various centers (see Table S2, Supplementary Materials), based on their g-values. Thus, the narrow line at g ~ 2.005 (A) was tentatively attributed to the perpendicular component of the g parameter of O^−^ centers (the O^−^(II) species reported by D’Arienzo et al. [42]). A shoulder at g ~ 2.004 and a low-intensity feature around g = 2.00 could be fitted with a rhombic spectrum (B) with parameters close to those of surface-adsorbed CO_2_^−^ ions [43]. The broad line (~10 mT) at g ~ 2.001 (C) was observed in the T400W spectrum in the whole temperature range, but it did not appear in the T400B spectrum (Figure 6b). This line was attributed to impurity ions such as Fe^3+^ segregated at the TiO_2_ nanoparticles surface, forming multinuclear clusters [44]. During the chemical reduction treatment, these surface Fe^3+^ ions could capture electrons and turn into the EPR silent Fe^2+^ ions, explaining why the C signal was not observed in the T400B spectrum. Based on their EPR spectrum intensity, the concentration of native iron impurities was below 10^−3^%. The two weak lines at higher magnetic fields correspond to the Ti^3+^(II) centers in anatase [45] (see Table 2).

As seen in Figure 5b, the T400B spectrum is dominated by a very broad and intense line at g ~1.94 (~1250 mT), associated with Ti^3+^ ions in a disordered environment at the surface or in the subsurface region of the anatase nanoparticles [46]. However, unlike the case of the other black samples, several significant lines appeared in the spectrum region around g ~ 2 (Figure 6b). The most intense line belongs to the F^+^ centers. The A line is still present, while the B line assigned to adsorbed CO_2_ and the C line associated with surface impurity clusters are not observed anymore. The narrow line (~0.6 mT) at g ~ 2.0006 (D), visible in the whole temperature range, was associated with conduction electrons in anatase [38]. Based on their EPR intensity, we could estimate that the concentration of the Ti^3+^ centres in the T400B sample was more than two orders of magnitude higher than the concentration of the hole centers such as the F^+^ and A centers. 

**Figure 7:**
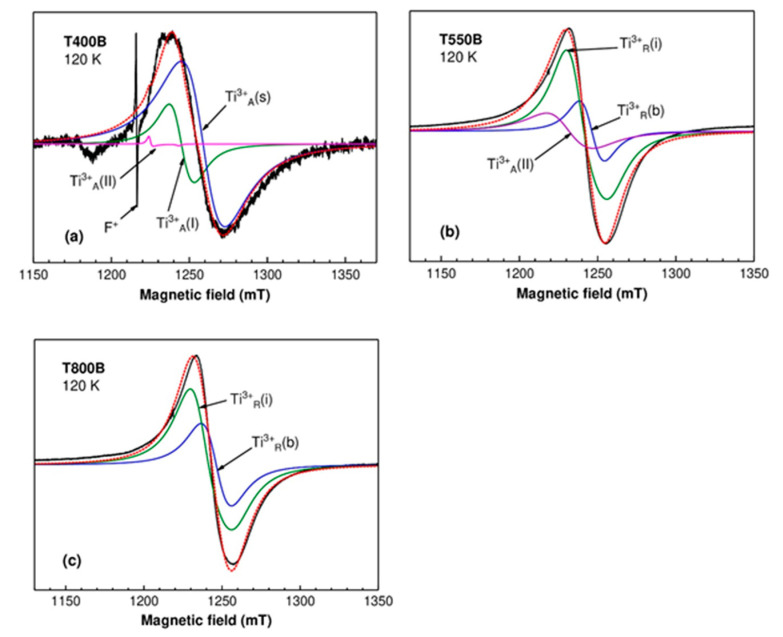
Experimental (solid black line) spectra of the T400B (**a**), T550B (**b**), and T800B (**c**) samples at 120 K. The simulated spectra (red dotted lines) are the sum of the calculated spectra of the different Ti^3+^ centers.

Figure 7a displays a deconvolution of the intense broad line (~33 mT) from the T400B spectrum into components associated with Ti^3+^ centers in anatase, with parameters given in Table 2. The best fit was obtained for a combination of (90 ± 3)% surface Ti^3+^_A_(s) centers and (10 ± 3)% bulk Ti^3+^_A_(I) centers. The Ti^3+^_A_(II) centers were included because of their presence in the T400W sample and to account for the lower field shoulder of the broad line. However, their concentration should be less than 2% for a reasonably good fit. 

In the case of the T550B sample, the presence of both rutile and anatase phases had to be taken into consideration for the deconvolution of the broad (~23 mT) and asymmetric line at g ~ 1.963 (~1240 mT) (Figure 7b). The possible contributors were found to be the Ti^3+^_A_(II) centers in anatase, the Ti^3+^ centers in regular cation sites Ti^3+^_R_(b), and interstitial sites Ti^3+^_R_(i) in bulk rutile [46]. However, the EPR parameters of the substitutional Ti^3+^_R_(b) [46] and surface Ti^3+^_R_(s) [47] centers in rutile (see Table 2) are close enough that, for the very broad linewidth used in the simulations (13–15 mT), their spectra cannot be distinguished. The same situation was encountered for the Ti^3+^_A_(I) centers in anatase and Ti^3+^_R_(b) centers in rutile. Therefore, the so-called “substitutional sites in bulk rutile” contribution could be partially due to Ti^3+^ ions at the surface of the rutile particles or in the bulk of the smaller anatase nanoparticles. 

The determination of the relative concentrations of the three centers in the T550B sample was not straightforward. Thus, the simulation in Figure 7b was obtained for a combination of 26% Ti^3+^_A_(II), 12% Ti^3+^_R_(b), and 62% Ti^3+^_R_(i) centers. However, we obtained reasonably good fits for a wide range of relative concentrations of the three centers, with the Ti^3+^_A_(II) proportion as low as 10% or the Ti^3+^_R_(b) proportion less than 3%. The Ti^3+^_R_(i) proportion was dominant in all cases (larger than 55%).

Figure 7c displays the deconvolution of the T800B broad line (~24 mT), centered at g ~ 1.961 (~1245 mT), into components assigned to Ti^3+^ ions in rutile. The best fit was obtained for the combination of (70 ± 3)% interstitial Ti^3+^_R_(i) centers and (30 ± 3)% substitutional Ti^3+^_R_(b) centers in bulk rutile. As in the case of the T550B sample, the presence of surface Ti^3+^_R_(s) centers cannot be dismissed.

A comparison of the three black samples shows that, in the anatase sample, most of the Ti^3+^ ions are localized on the nanoparticles surface, while for the other two samples, where rutile was the dominant or only phase, the majority of the Ti^3+^ ions are found in bulk interstitial or substitutional sites [42].

### 3.3. Photodegradation and Mineralization of Amoxicillin

In order to determine the photocatalytic efficiency of the black TiO_2_ in the degradation of AMX, three types of experiments were performed. The first one was designed to evaluate the catalytic activity of the six samples in the dark. For the adsorption of amoxicillin on photocatalysts in the dark, there was only a 5% change in AMX concentration. In the second experiment, AMX was exposed to visible light irradiation without adding a catalyst, and no significant change in the concentration of the drug was observed due to self-degradation. In the third experiment, the photocatalytic activities of the six photocatalysts were evaluated. In addition, the AMX mineralization was investigated by measuring the total organic carbon (TOC).

**Figure 8:**
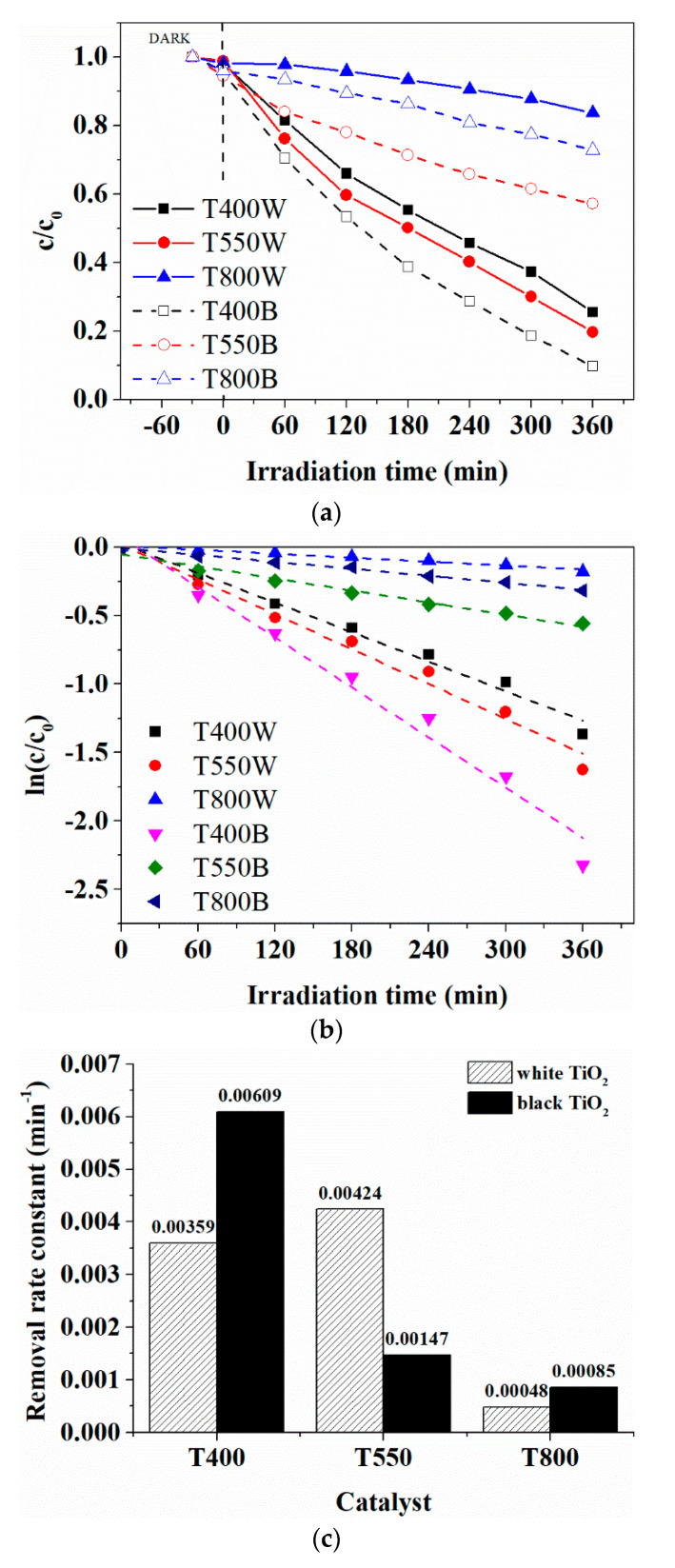
(**a**) Evaluation of the visible-light-driven photocatalytic activity of the original white TiO_2_ and black TiO_2_ prepared through NaBH_4_ reduction. (**b**) A plot of ln(c/c_0_) versus irradiation time for the photocatalytic degradation of amoxicillin at different catalysts. (**c**) First-order kinetics rate constant of amoxicillin removal by visible simulated irradiation over original white and black titanium oxide photocatalysts.

**Figure 9:**
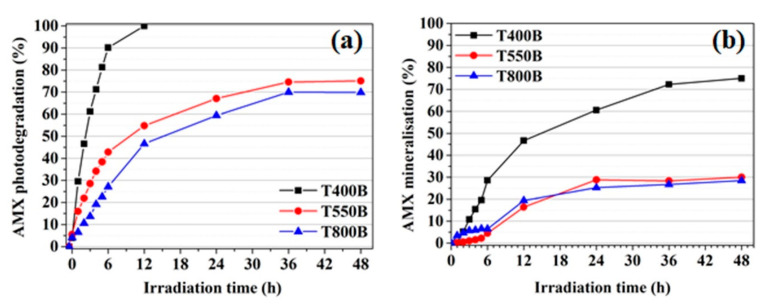
(**a**) The c/c_0_ versus irradiation time curves of black TiO_2_ for long-term AMX photodegradation. (**b**) The AMX mineralization efficiency for long-term degradation over black TiO_2_ photocatalysts.

Figure 8a shows that T400B is a highly efficient photocatalyst, leading to complete AMX degradation and 30% mineralization (Figure 9b) after six hours of visible light irradiation. For the other samples, after the same irradiation time, the AMX degradation efficiency of 43% for T550B and only 27% for T800B was achieved, whereas the mineralization was only 5–6%. It should be noted that only the T400B sample showed a degradation efficiency ~15% higher than its white counterpart (T400W). This result is consistent with the EPR results and justified by the presence of surface Ti^3+^ defects in a concentration much higher than in the other black TiO_2_ powders. This phenomenon occurred because upon NaBH_4_ reduction, the defects such as Ti^3+^ and oxygen vacancies were introduced at the surface of the TiO_2_ nanoparticles, forming black TiO_2_ with intense light absorption in the visible range due to the narrowing of the TiO_2_ bandgap from 3.32 to 2.92 eV. Therefore, the T400B material has the best photodegradation and mineralization efficiencies of amoxicillin due to its specific materials properties such as, e.g., large specific surface area, a small crystallite size of 12 nm, anatase structure, and uniformity of particle size of 325 nm in dispersion. Although the poor visible light performance of the samples T800W and T800B is associated with the rutile phase being less active than the anatase, the low BET surface area and large nanoparticles size (Table 1) contributed to the low photocatalytic activity as well. A reversed behavior of the photocatalytic activity, consisting of higher efficiency of the white sample compared to the activity of the black sample, was observed for the powder containing both anatase (85%) and rutile (15%), mainly due to the synergetic effect between anatase and rutile, as also observed by Ma and Chen [48].

In order to observe the complete degradation (Figure 9a) and mineralization (Figure 9b) of AMX, long-term studies of up to 48 h were performed only on the black TiO_2_ catalysts. As a result, a levelling-off trend toward mineralization efficiencies of ~30% was observed for the T550B and T800B materials, while the T400B material had a 75% mineralization efficiency of AMX at 48 h of irradiation.

## 4. Conclusions

We have investigated the effect of chemical reduction with NaBH_4_ on the photocatalytic activity of three samples of white TiO_2_ prepared by a similar sol-gel-based technique and annealed at three different temperatures: 400 °C, 550 °C and 800 °C. According to the XRD and (HR)TEM investigations, the 400 °C white and black (chemically reduced) samples consist of anatase nanoparticles with an average crystallite size of 15 nm and 12 nm, respectively. The 550 °C sample consists of a mixture of both anatase (16%) and rutile (84%) phases, with the average crystallite size for anatase being three times larger than for the 400 °C samples, while for the rutile phase, it decreases from 127 nm to 75 nm after chemical reduction. The 800 °C white and black samples consist of rutile particles with an average crystallite size of 130 nm. The chemical reduction induced a net decrease of the direct band gap: from 3.32 eV to 2.92 eV for the 400 °C samples and from 3.08 eV to 2.96 eV for the 550 °C. The EPR investigations revealed the main effect of the chemical reduction treatment, namely the formation of a large quantity of Ti^3+^ defects. The quantitative analysis of the EPR spectra of the three black samples shows that in the 400 °C anatase sample, ~90% of the Ti^3+^ ions are localized on the nanoparticles surface, while for the other two samples, where rutile was the dominant or only phase, the majority of the Ti^3+^ ions are found in bulk interstitial or substitutional sites. The black anatase sample exhibits the highest photocatalytic degradation and mineralization activity of amoxicillin, due to the anatase structure, the small size of the nanoparticles, and the localization of the Ti^3+^ defects predominantly on the surface of the photocatalyst.

## Figures and Tables

**Table 1:** Specific surface area (S_BET_), average pore volume and pore diameter, and average particle size for TiO_2_ samples.

Samples	S_BET_ (m^2^/g)	Average Pore Volume (cm^3^/g)	Average Pore Diameter (nm) ^1^	Average Particle Size (nm) ^2^	Average Aggregate Particle Size (nm) ^3^
TiO_2_ sol-gel	266.9	0.2352	4.58	22.48	-
T400W	89.08	0.1631	7.49	67.35	1911
T400B	90.21	0.1460	6.32	66.5	333.3
T550W	5.64	0.0095	13.15	1062	828.8
T550B	7.099	0.0113	14.94	845	376.5
T800W	2.41	0.0011	2.15	2484	457.7
T800B	3.86	0.0058	8.38	1552	401.9

^1^ calculated from the Barrett, Joyner, and Halenda (BJH) equation from the analysis of the desorption curve. ^2^ provided by the N_2_ adsorption–desorption measurement. ^3^ obtained by the DLS measurement in aqueous dispersion.

**Table 2:** EPR parameters of the Ti^3+^ centers in anatase and rutile.

Centers	Assignment	g Values	Reference
Ti^3+^_A_(I)	Ti^3+^ in bulk anatase	g_x_ = g_y_ = 1.9640 g_z_ = 1.9495	[45]
Ti^3+^_A_(II)	Ti^3+^ in bulk anatase	g_x_ = g_y_ = 1.992 g_z_ = 1.962	[45] [46]
Ti^3+^_A_(s)	Ti^3+^ in disordered environment (surface) in anatase	g = 1.940 ± 0.004	This work
Ti^3+^_R_(b)	Ti^3+^ in regular cation site in bulk rutile	g_x_ = 1.969 g_y_ = 1.960 g_z_ = 1.949	[46]
Ti^3+^_R_(i)	Ti^3+^ in interstitial site in bulk rutile	g_x_ = 1.9787 g_y_ = 1.9750 g_z_ = 1.9424	[46]
Ti^3+^_R_(s)	Ti^3+^ in surface or subsurface sites (dehydrated/hydrated surface) in rutile	g_x_ = 1.970/1.973 g_y_ = 1.961 g_z_ = 1.948	[47]

## Data Availability

Not applicable.

## Supplementary Materials

The following supporting information can be downloaded at https://www.mdpi.com/article/10.3390/nano12152563/s1. Table S1: The crystalline phase, average crystallite size, and lattice parameters of the TiO_2_ samples. Figure S1: (a) Electron diffraction pattern obtained by selecting the large nanoparticles from Figure 2b; the intense diffraction spots correspond to the (110) and (011) planes of rutile structure; (b) electron diffraction pattern obtained by selecting the small nanoparticles from Figure 2b; the most intense diffraction spots correspond to the (020), (015), (112), and (211) crystallographic planes of anatase structure. Figure S2: EDS spectra of (a) T400W, (b) T400B, (c) T550W, (d) T550B, (e) T800W, (f) T800B with the characteristic peaks of the Ti and O constituents. Small peaks associated with traces of Na (below the 1.5% quantification limit) are observed in the EDS spectra of T400B and T550B. The C and Cu lines are due to scattering electrons on the TEM grid. Figure S3: Hydrodynamic particle size distribution in DLS measurements (average particle size and PDI = polydispersive index are listed in parentheses). Figure S4: Two hysteresis loops: H3 hysteresis for T400W (a) and T400B (d), and H1 hysteresis for T550W (b), T800W (c), T550B (e), and T800B (f) from nitrogen adsorption–desorption measurements. Figure S5: Band-gap (E_g_) measurements from DRS data using the Kubelka–Munk function. Table S2: EPR parameters determined for the paramagnetic centers with spectra in the g ~ 2 region, observed in the T400W and T400B samples.
