# Supplementary material for: Visible-Light-Active Black TiO2 Nanoparticles with Efficient Photocatalytic Performance for Degradation of Pharmaceuticals
Source: Nanomaterials (Basel). 2022 Jul 26;12(15):2563. doi: 10.3390/nano12152563 (PMC9330099; doi:10.3390/nano12152563)
Supplement: Supplementary file 1 [file nanomaterials-12-02563-s001.zip › nanomaterials-1844454-supplementary.pdf]

Table S1. The crystalline phase, average crystallite size and lattice parameters of the TiO_2 samples.

Samples	Colour	Synthesis method	Crystalline phase	Crystalite size (nm)	Lattice constant (Å)	
					a	c
T400W	White	Sol-gel, 400°C	Anatase: 100%	14.9	3.790	9.507
T550W	White	Sol-gel, 550°C	Anatase: 16%	45.1	3.784	9.513
			Rutile: 84%	126.8	4.593	2.959
T800W	White	Sol-gel, 800°C	Rutile: 100%	130.4	4.593	2.959
T400B	Black	Chemical reduction with $NaBH_4$, 350°C	Anatase: 100%	11.9	3.800	9.511
T550B	Black		Anatase: 15 %	38.5	3.784	9.506
			Rutile: 85 %	74.8	4.594	2.959
T800B	Black		Rutile: 100%	130.7	4.593	2.959

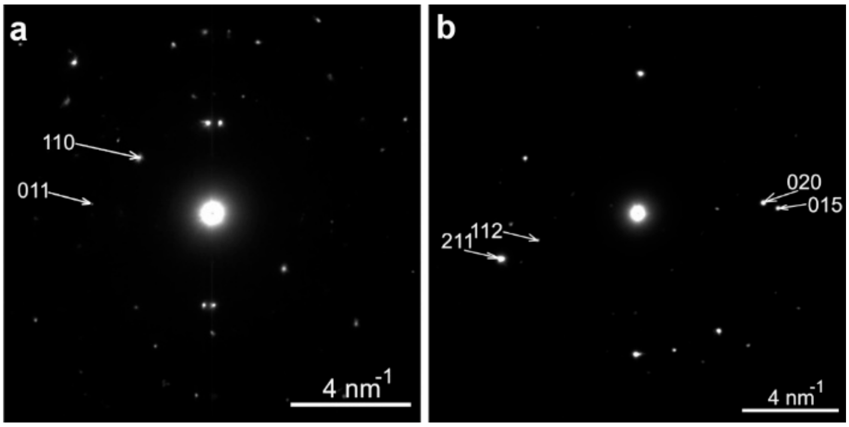

Figure S1. (a) Electron diffraction pattern obtained by selecting the large nanoparticles from Fig. 2b; the intense diffraction spots correspond to the (110) and (011) planes of rutile structure; (b) electron diffraction pattern obtained by selecting the small nanoparticles from Fig. 2b; the most intense diffraction spots correspond to the (020), (015), (112) and (211) crystallographic planes of anatase structure.

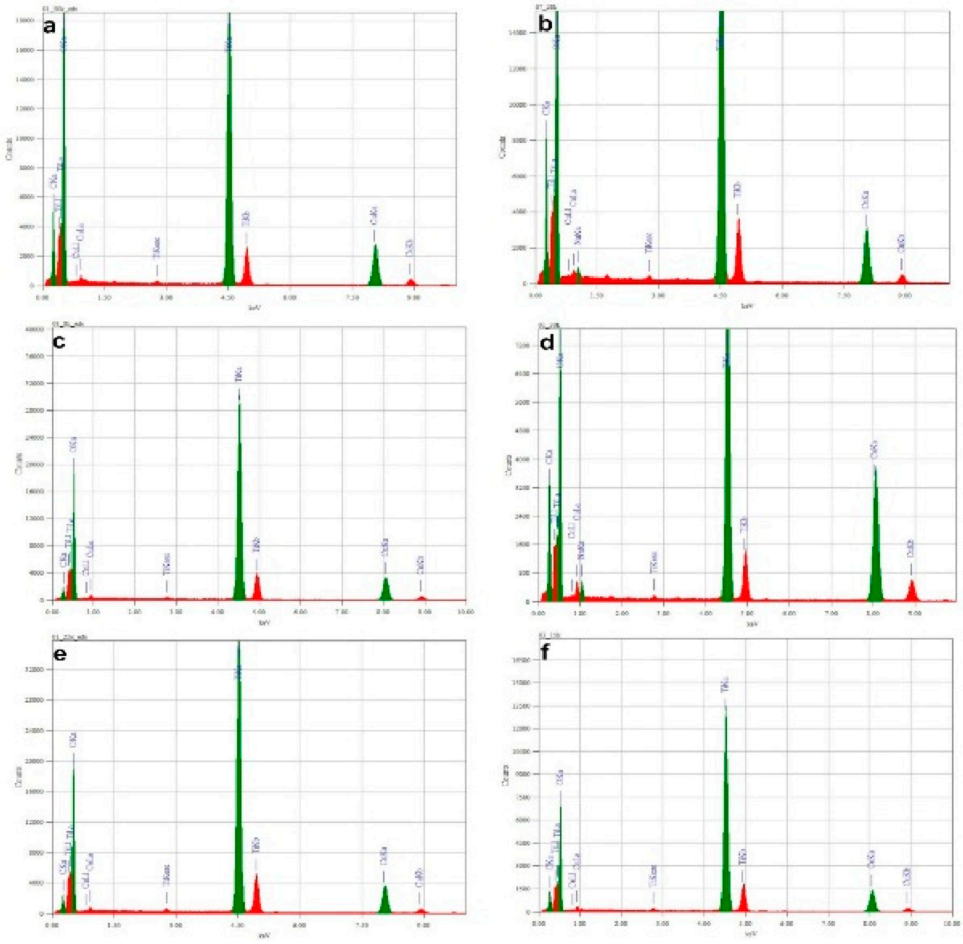

Figure S2. EDS spectra of (a) T400W, (b) T400B, (c) T550W, (d) T550B, (e) T800W, (f) T800B with the characteristic peaks of the Ti and O constituents. Small peaks associated with traces of Na (below the 1.5% quantification limit) are observed in the EDS spectra of T400B and T550B. The C and Cu lines are due to scattering electrons on the TEM grid.

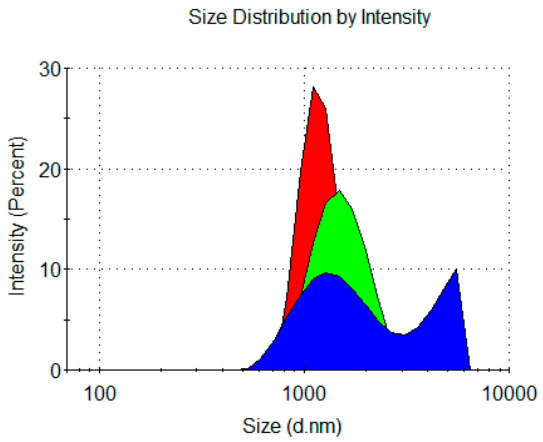

T400W (1.835 μm, 1.855 μm, 2.042 μm)

(PDI=0.3-0.48)

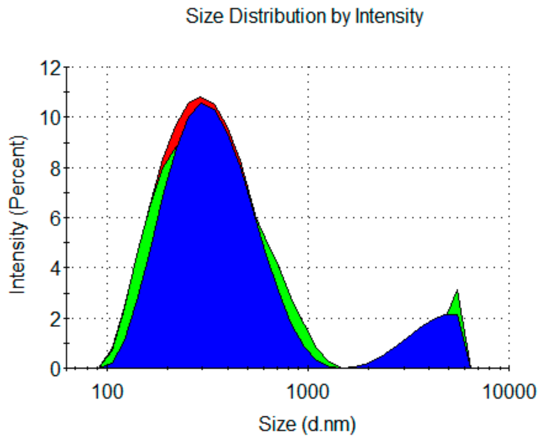

T400B (325 nm, 330 nm, 344 nm)

(PDI=0.34-0.39)

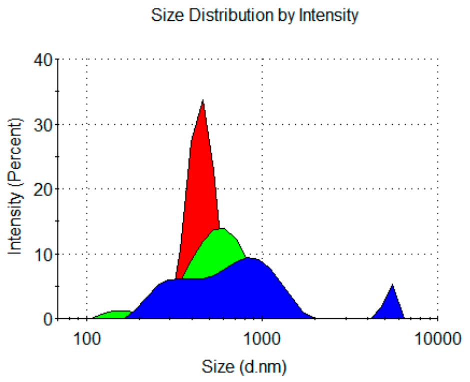

T550W (712 nm, 772 nm, 1002 nm)

(PDI=0.58-0.68)

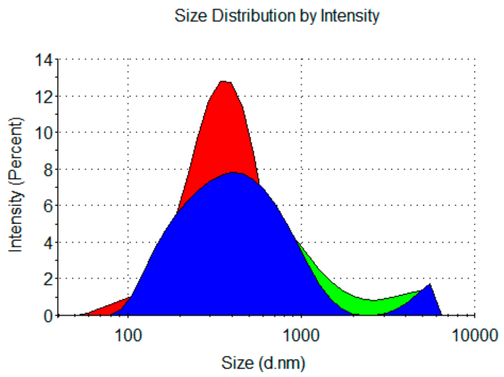

T550B (359 nm, 379 nm, 391 nm)

(PDI=0.36-0.40)

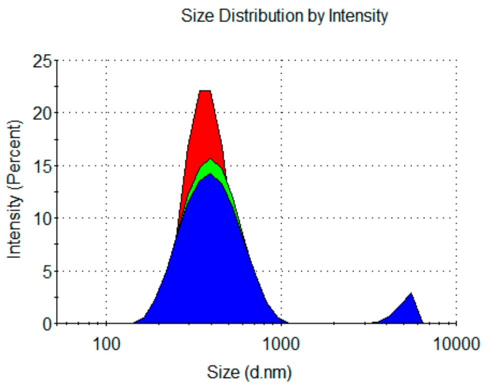

T800W (435 nm, 446 nm, 491 nm)

(PDI=0.28-0.36)

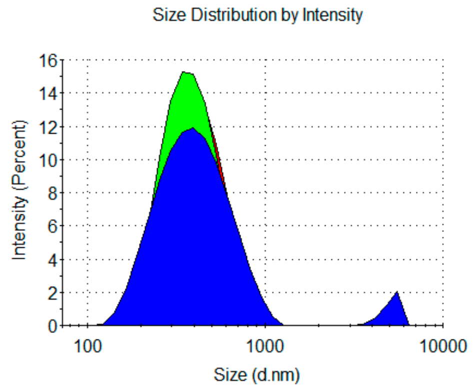

T800B (396 nm, 399 nm, 410 nm)

(PDI=0.24-0.30)

Figure S3. Hydrodynamic particle size distribution in DLS measurements (average particle size and PDI = polydispersive index are listed in parentheses)

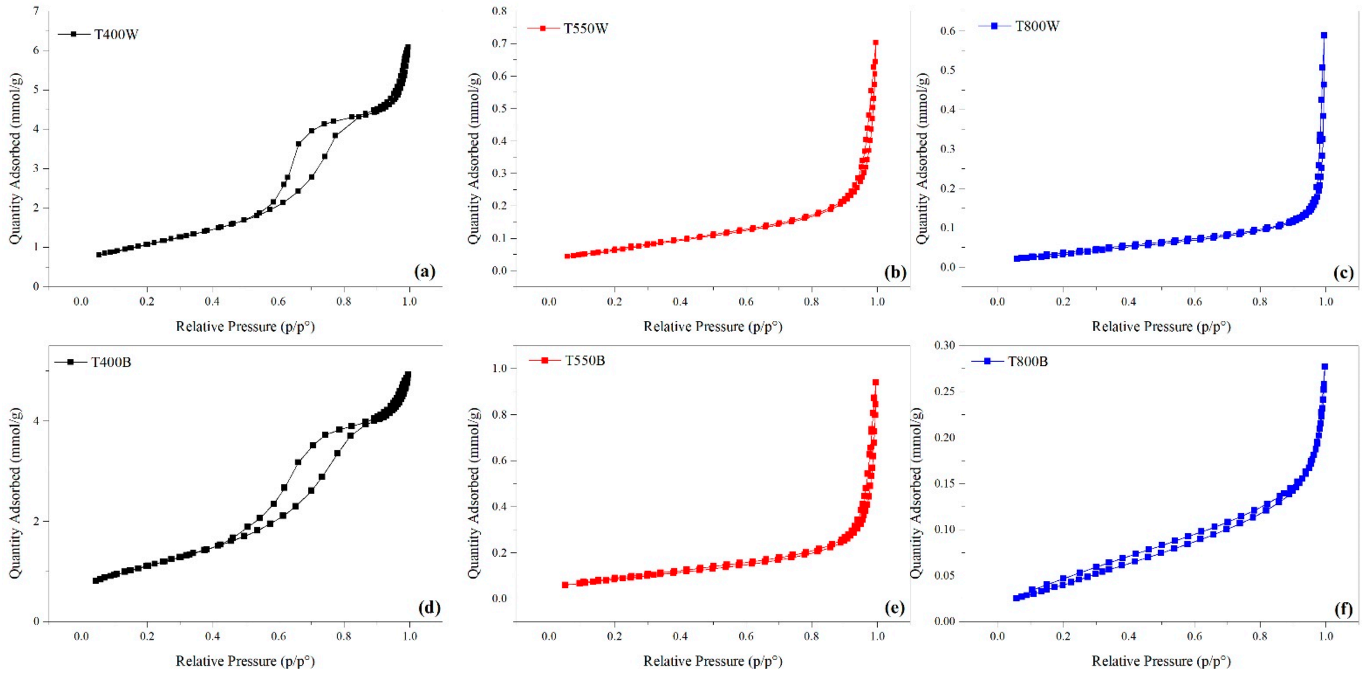

Figure S4. Two hysteresis loops H3 hysteresis for T400W(a) and T400B (d), and H1 hysteresis for T550W(b), T800W(c), T550B (e) and T800B (f) from nitrogen adsorption-desorption measurements.

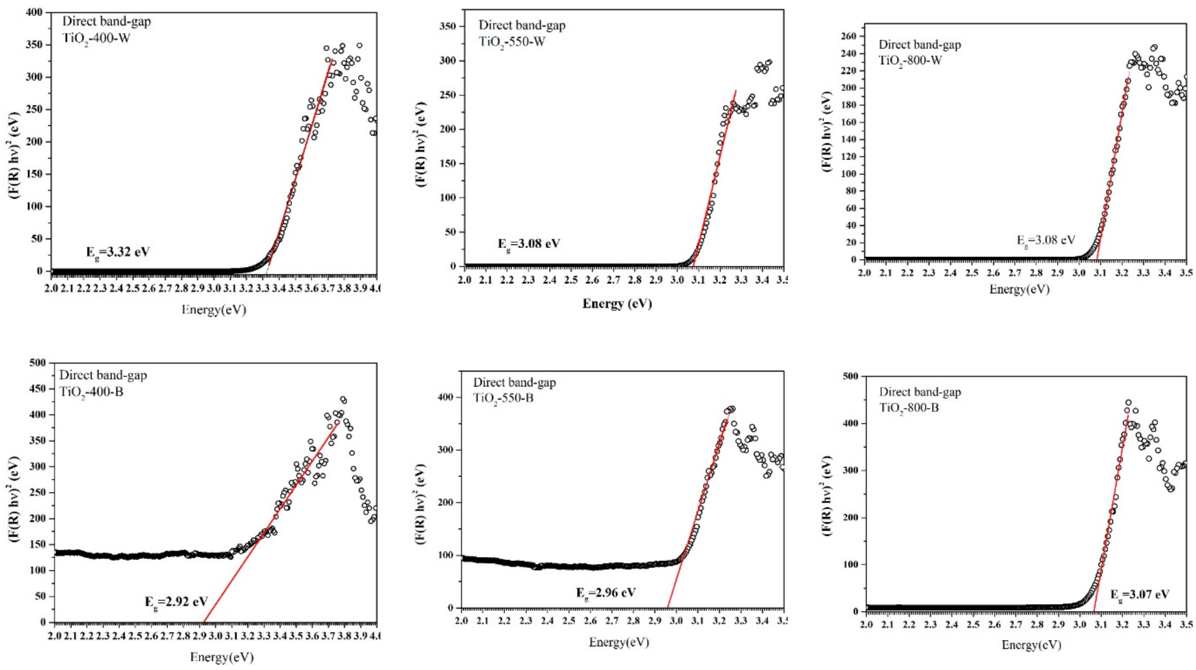

Figure S5. Band-gap (E_g) measurements from DRS data using the Kubelka-Munk function.

Table S2. EPR parameters determined for the paramagnetic centres with spectra in the g ~ 2 region, observed in the T400W and T400B samples.

Centres	Assignment	g values
F^+	Electron trapped at an oxygen vacancy	$g = 2.0028 \pm 0.0003$
A	Hole center, oxygen related (possibly O^- [(a)])	$g_x = g_y = 2.0053 \pm 0.0002$<br>$g_z = 1.998 \pm 0.0005$
B	Surface adsorbed CO_2^-	$g_x = 2.0032 \pm 0.0005$<br>$g_y = 2.0008 \pm 0.0005$<br>$g_z = 1.997 \pm 0.0003$
C	Multinuclear paramagnetic ion (e.g. Fe^{3+}) clusters at TiO_2 nanoparticles surface	$g = 2.001 \pm 0.001$
D	Conduction electrons	$g = 2.0006 \pm 0.0002$

[a)] M. D'Arienzo, J. Carbajo, A. Bahamonde, M. Crippa, S. Polizzi, R. Scotti, L. Wahba, F. Morazzoni, Photogenerated Defects in Shape-Controlled TiO_2 Anatase Nanocrystals: A Probe To Evaluate the Role of Crystal Facets in Photocatalytic Processes, J. Am. Chem. Soc. 133 (2011) 17652–17661. https://doi.org/10.1021/ja204838s.

FIGURES CAPTION

Figure S1 (a) Electron diffraction pattern obtained by selecting the large nanoparticles from Fig. 2b; the intense diffraction spots correspond to the (110) and (011) planes of rutile structure; (b) electron diffraction pattern obtained by selecting the small nanoparticles from Fig. 2b; the most intense diffraction spots correspond to the (020), (015), (112) and (211) crystallographic planes of anatase structure

Figure S2 EDS spectra of (a) T400W, (b) T400B, (c) T550W, (d) T550B, (e) T800W, (f) T800B with the characteristic peaks of the Ti and O constituents. Small peaks associated with traces of Na (below the 1.5% quantification limit) are observed in the EDS spectra of T400B and T550B. The C and Cu lines are due to scattering electrons on the TEM grid.

Figure S3 Hydrodynamic particle size distribution in DLS measurements (average particle size and PDI = polydispersive index are listed in parentheses)

Figure S4 Two hysteresis loops H3 hysteresis for T400W(a) and T400B (d), and H1 hysteresis for T550W(b, T800W(c), T550B (e) and T800B (f) from nitrogen adsorption-desorption measurements

Figure S5 Band-gap (E_g) measurements from DRS data using the Kubelka-Munk function

TABLES CAPTION

Table S1 The crystalline phase, average crystallite size and lattice parameters of the TiO_2 samples

Table S2 EPR parameters determined for the paramagnetic centres with spectra in the g ~ 2 region, observed in the T400W and T400B samples
